# Arsenic Exposure from Drinking Water and QT-Interval Prolongation: Results from the Health Effects of Arsenic Longitudinal Study

**DOI:** 10.1289/ehp.1205197

**Published:** 2013-02-05

**Authors:** Yu Chen, Fen Wu, Faruque Parvez, Alauddin Ahmed, Mahbub Eunus, Tyler R. McClintock, Tazul Islam Patwary, Tariqul Islam, Anajan Kumar Ghosal, Shahidul Islam, Rabiul Hasan, Diane Levy, Golam Sarwar, Vesna Slavkovich, Alexander van Geen, Joseph H. Graziano, Habibul Ahsan

**Affiliations:** 1 Department of Population Health, and; 2 Department of Environmental Medicine, New York University School of Medicine, New York University, New York, New York, USA; 3 Department of Environmental Health Sciences, Mailman School of Public Health, Columbia University, New York, New York, USA; 4 U Chicago Research Bangladesh Ltd., Dhaka, Bangladesh; 5 New York University School of Medicine, New York University, New York, New York, USA; 6 Lamont-Doherty Earth Observatory of Columbia University, Palisades, New York, USA; 7 Departments of Health Studies, Medicine and Human Genetics and Comprehensive Cancer Center, The University of Chicago, Chicago, Illinois, USA

**Keywords:** arsenic, Bangladesh, cardiovascular disease, electrocardiogram, heart rate–corrected QT interval, environmental exposure

## Abstract

Background: Arsenic exposure from drinking water has been associated with heart disease; however, underlying mechanisms are uncertain.

Objective: We evaluated the association between a history of arsenic exposure from drinking water and the prolongation of heart rate–corrected QT (QTc), PR, and QRS intervals.

Method: We conducted a study of 1,715 participants enrolled at baseline from the Health Effects of Arsenic Longitudinal Study. We assessed the relationship of arsenic exposure in well water and urine samples at baseline with parameters of electrocardiogram (ECG) performed during 2005–2010, 5.9 years on average since baseline.

Results: The adjusted odds ratio (OR) for QTc prolongation, defined as a QTc ≥ 450 msec in men and ≥ 460 msec in women, was 1.17 (95% CI: 1.01, 1.35) for a 1-SD increase in well-water arsenic (108.7 µg/L). The positive association appeared to be limited to women, with adjusted ORs of 1.24 (95% CI: 1.05, 1.47) and 1.24 (95% CI: 1.01, 1.53) for a 1-SD increase in baseline well-water and urinary arsenic, respectively, compared with 0.99 (95% CI: 0.73, 1.33) and 0.86 (95% CI: 0.49, 1.51) in men. There were no apparent associations of baseline well-water arsenic or urinary arsenic with PR or QRS prolongation in women or men.

Conclusions: Long-term arsenic exposure from drinking water (average 95 µg/L; range, 0.1–790 µg/L) was associated with subsequent QT-interval prolongation in women. Future longitudinal studies with repeated ECG measurements would be valuable in assessing the influence of changes in exposure.

Epidemiologic studies have linked arsenic exposure from drinking water to cardiovascular disease (CVD) (Chen CJ et al. 1996; Chen Y et al. 2011a; Chiou et al. 1997; Tseng et al. 2003) and to heart disease in particular (Chen CJ et al. 1996; Chen Y et al. 2011a; Tseng et al. 2003). However, the underlying mechanism by which arsenic may lead to heart disease is unclear.

The QT interval on an electrocardiogram (ECG) is defined as the time from the onset of the QRS complex to the end of the T wave. It represents the duration of ventricular electrical systole, including depolarization and repolarization. Its prolongation indicates nonuniform recovery of myocardial excitability and has been shown to lower the ventricular fibrillation threshold and increase susceptibility to ventricular arrhythmia and sudden cardiac death (Algra et al. 1991; Straus et al. 2006). QT-interval prolongation has been associated with an increased risk of cardiovascular and all-cause mortality in a broad range of clinical populations as well as in healthy subjects in population-based studies (Goldberg et al. 1991; Okin et al. 2000). Arsenic has been demonstrated to prolong the QT interval both in animal studies (Ficker et al. 2004; Sun et al. 2006) and in cases of acute arsenic poisoning (Chiang et al. 2002; Hall and Harruff 1989). Clinical studies consistently demonstrate that arsenic trioxide, used to treat acute promyelocytic leukemia, induces QT prolongation, torsades de pointes (a life-threatening polymorphic ventricular tachycardia), and sudden death (Ohnishi et al. 2000; Westervelt et al. 2001). Several studies have reported positive associations between arsenic exposure from drinking water and ECG abnormalities, including QT-interval prolongation and QT dispersion (Ahmad et al. 2006; Mordukhovich et al. 2009; Mumford et al. 2007; Wang et al. 2009; Yildiz et al. 2008). However, small sample sizes (Ahmad et al. 2006; Yildiz et al. 2008) and the use of ecological measures of exposure (Ahmad et al. 2006; Wang et al. 2009) have limited the interpretation of study results. In addition, all previous studies measured arsenic exposure at one point in time, without information on longitudinal exposure.

It has been estimated that 13 million Americans have been exposed to drinking water with arsenic concentrations of 10–50 µg/L (Gianfrancesco et al. 2003). In Bangladesh, an estimated 57 million people have been chronically exposed to arsensic from drinking groundwater with arsenic concentrations exceeding the World Health Organization standard (10 µg/L) (British Geological Survey 2007). In 2000, we established a large prospective cohort study of > 20,000 adults in Araihazar, Bangladesh, to evaluate the health effects of arsenic exposure. More than 90% of the cohort has been exposed to drinking water with arsenic concentrations of 0.1–300 µg/L (including > 47% exposed to 0.1–50 µg/L), providing us with a unique opportunity to study cardiovascular outcomes in a population exposed to arsenic at moderate levels. In the present study, we estimated associations between baseline arsenic exposure and subsequent ECG abnormalities, including prolongation of heart rate–corrected QT (QTc), PR, and QRS intervals in 1,715 cohort members.

## Materials and Methods

*Study population.* The Health Effects of Arsenic Longitudinal Study (HEALS) is an ongoing population-based, prospective cohort study in Araihazar, Bangladesh. Details of the study methodologies have been presented elsewhere (Ahsan et al. 2006a). Briefly, we recruited 11,746 residents 18–75 years of age (original cohort) in 2000. HEALS was expanded to include an additional 8,287 participants (expansion cohort) in 2007–2008. The overall response rate was 97%. The cohort is being actively followed with personal visits at 2-year intervals, which include a physical examination, collection of urine samples, and a structured interview conducted by trained physicians following the same procedures used in the baseline interview. Interim health surveys have been conducted every 6 months between the biennial follow-up visits. A field clinic was established exclusively for the cohort participants to receive medical diagnoses and treatments and facilitate the follow-up (Ahsan et al. 2006a). Oral informed consent was provided by study participants, and study procedures were approved by the ethics committee of the Bangladesh Medical Research Council and the institutional review boards of Columbia University and the University of Chicago.

*Measurements of arsenic exposure.* Information on arsenic exposure was prospectively collected beginning with concentrations measured in drinking water and urine at baseline recruitment, and in follow-up urine samples collected every 2 years. At baseline, water samples from 10,971 contiguous wells were collected in 20 mL polyethylene scintillation vials, which were first rinsed several times with groundwater. The samples were acidified to 1% with high-purity Optima hydrochloric acid (Fisher Scientific, Pittsburgh, PA, USA) for at least 48 hr before analysis. Total arsenic concentration was analyzed by high-resolution inductively-coupled plasma mass spectrometry, with a detection limit of < 0.2 µg/L. Further details on field sampling and laboratory analysis procedures are described elsewhere (Cheng et al. 2004; Van Geen et al. 2005). The long-term reproducibility determined from consistency standards included with each run averaged 4% (1 σ) in the 40–500 µg/L range.

Spot urine samples were collected in 50 mL acid-washed tubes from 95.6%, 94.5%, and 91.2% of the original cohort participants at baseline and the first and second follow-up visits, respectively. Total arsenic concentration was measured by graphite furnace atomic absorption, using a PerkinElmer AAnalyst 600 graphite furnace system (PerkinElmer, Waltham, MA, USA) with a detection limit of 2 μg/L, as previously described (Nixon et al. 1991). Urinary creatinine was analyzed by a colorimetric Sigma diagnostics kit (Sigma, St. Louis, MO, USA) for adjustment of urinary total arsenic concentration (Slot 1965).

*ECG measurements and evaluation.* Participants in the present analysis were 1,715 cohort members referred for ECG examinations because of high blood pressure or symptoms of heart disease (including chest pain, shortness of breath, irregular heartbeat, and palpitations) identified during a biennial follow-up study visit, an interim health survey, or a visit to the field clinic for medical treatment. These participants were referred to one of three trained field clinic physicians, who were blinded to arsenic-exposure information, for further evaluation and diagnostic confirmation followed by treatment and referral to the only local hospital in the study area as appropriate. Standard 12-lead resting ECGs were performed using a Bionet Cardiocare 2000 device (Bionet America Inc., Tustin, CA, USA). All ECGs were processed using the Dalhousie ECG program (Rautaharju et al. 1990). The QT interval was measured from the beginning of the QRS complex to the end of the T wave, and was corrected for heart rate using the Bazzet formula (QTc = QT ÷ [60/heart rate]^1/2^) (Bazett 1920). The PR interval was measured from the beginning of the P wave to the beginning of the QRS complex, and the QRS interval was measured from the beginning of the QRS complex to the end of the QRS complex. Prolongation was defined as a QT interval ≥ 450 msec in men and ≥ 460 msec in women (Rautaharju et al. 2009), a PR interval > 200 msec (Cheng et al. 2009), and a QRS interval ≥ 120 msec (Dhar et al. 2008). ECGs were conducted between 2005 and 2010, with an average time since baseline of 5.9 years.

*Statistical analyses.* We first conducted descriptive analyses to compare arsenic exposure data and demographic and lifestyle characteristics between the present study population and the overall cohort, and between participants with and without QTc prolongation in the study population.

We used unconditional logistic regression to estimate the odds ratios (ORs) for QTc prolongation in relation to quartiles of baseline well-water and urinary arsenic. We first adjusted for sex and age (years) (model 1); we then additionally adjusted for educational attainment (years), baseline body mass index (BMI; kilograms per meter squared), and baseline smoking status (never, past, and current) (model 2) (Ahsan et al. 2006b; Argos et al. 2007). Data for the full cohort suggested little change in BMI, educational attainment, and smoking status over time; therefore, these variables were not measured again at the time of ECG evaluation. Because arsenic exposures may have changed from baseline levels in some participants, we adjusted the final model (model 3) for changes in urinary arsenic between visits. We assumed that urinary arsenic levels were unchanged from baseline among expansion cohort participants (*n* = 273) who did not have follow-up measurements because of the recency of their recruitment (1.8 years from baseline to ECG evaluation). For subjects in the original cohort, we adjusted for the difference in urinary arsenic between baseline and the first follow-up, and between the first and second follow-up, in 1,402 participants from the original cohort whose ECGs were performed after the second follow-up or within the 6 months before the second follow-up. For participants whose ECG was performed > 6 months before the second follow-up (*n* = 38), we adjusted for the difference between baseline and the first follow-up only. Separate dummy variables for changes in urinary arsenic were created for participants who did not have urine arsenic concentrations measured at the first (*n* = 15) or second follow-up visit (*n* = 28) under a “missing at random” assumption. Linear regression models were also conducted to estimate associations between arsenic exposure and QTc as a continuous dependent variable, with the same adjustments as in the logistic regression models.

The literature suggests that women have longer average QTc intervals compared with men, and therefore we conducted stratified analysis to estimate associations of arsenic exposure with prolongation of QTc, PR, and QRS intervals separately in men and women, similar to previous studies (Benoit et al. 2005; Fukui et al. 2003; Giunti et al. 2007). ORs for QTc, PR, and QRS prolongation in relation to 1-SD increases in baseline well-water and urinary arsenic were also estimated.

We examined the assumption of a linear effect of arsenic exposure by including higher-order polynomial terms for arsenic exposure variables in the models, but found no indication of any nonlinear relation based on the significance of the β coefficient for the terms (data not shown). Additional analyses were conducted, including analyses restricted to the subpopulation who had a more complete exposure history because they used their baseline wells for ≥ 5 years prior to baseline (*n* = 1,142), analyses of associations with urinary arsenic in the sample collected closest to the ECG measurement, analyses excluding those with QRS of ≥ 120 msec [because the increased QRS duration may contribute to prolongation of the QT interval (Rautaharju et al. 2009)], and analyses of associations with heart rate–adjusted PR interval [PRa = PR + 0.26 (heart rate – 70) for participants < 60 years of age and PRa = PR + 0.42 (heart rate – 70) for those ≥ 60 years of age], with PRa prolongation defined as PRa > 205 msec (Soliman and Rautaharju 2012). In addition, because adjustment for creatinine might influence the relation between urinary arsenic and disease outcomes related to creatinine (Gamble and Liu 2005; Steinmaus et al. 2009), we adjusted urinary total arsenic for specific gravity (SG) to account for dilution, instead of adjusting for creatinine. Adjustment for SG was shown to be less affected by body size, socioeconomic status, and arsenic exposure compared with creatinine adjustment in a study carried out in Matlab, Bangladesh (Nermell et al. 2008). Urinary arsenic was adjusted to the overall mean SG value of 1.013 g/mL (range, 1.00–1.03 g/mL) in the study population, such that SG-adjusted urinary arsenic = [urinary arsenic × (1.013 – 1)] ÷ measured SG – 1. All analyses were performed using the SPSS version 19.0 software (IBM, New York, NY, USA). *p-*Values for trend (*p*_trend_) were estimated with arsenic exposure variables modeled as continuous variables. All tests were two sided, and *p* < 0.05 was considered significant.

## Results

Arsenic exposures and demographic and lifestyle characteristics were in general similar between the study population and the overall HEALS cohort, although the study population had a slightly higher proportion of women [see Supplemental Material, Table S1 (http://dx.doi.org/10.1289/ehp.1205197)].

The QTc prolongation was observed among 13.9% of the overall study participants (16.7% of women, and 9.1% of men) (Table 1). There was no consistent relationship between age and QTc prolongation. The proportions of never smokers, those with BMI ≥ 25.0, and those with elevated systolic or diastolic blood pressure (≥ 140 and 90 mmHg, respectively) were higher among cases than noncases. Average baseline well-water and urinary arsenic concentrations were higher among those with QTc prolongation than in noncases (106.0 µg/L vs. 92.8 µg/L for well-water arsenic, and 280.8 µg/L vs. 263.8 µg/L for urinary arsenic, respectively). Among members of the original cohort, total urinary arsenic decreased from baseline to the first follow-up by an average of 64.7 and 70.6 µg/g creatinine in those with QTc prolongation and noncases, respectively, whereas concentrations were relatively stable from the first to second follow-up in both groups. Cases of QTc prolongation had a higher heart rate, QTc, and QRS interval and a lower PR interval compared with noncases.

**Table 1:** Demographic, baseline lifestyle, and arsenic-exposure variables by QTc prolongation status.^a^

	QTc prolongation [n (%)]b		p-Valuec
	Yes (n = 237)	No (n = 1,474)	
Sex
Women	179 (75.5)	894 (60.7)	< 0.01
Men	58 (24.5)	580 (39.3)
Age at ECG exam (years)
20–29	3 (1.3)	55 (3.7)	< 0.01
30–39	57 (24.1)	353 (23.9)
40–49	84 (35.4)	507 (34.4)
50–59	85 (35.9)	410 (27.8)
60–77	8 (3.4)	149 (10.1)
Mean ± SD	46.1 ± 8.6	46.3 ± 9.9	0.79
Education (years)d
None	106 (44.7)	621 (42.2)	0.29
1–5	58 (24.5)	439 (29.8)
6–9	39 (16.5)	244 (16.6)
≥10	34 (14.3)	169 (11.5)
Mean ± SD	3.7 ± 4.0	3.6 ± 3.8	0.74
BMI (kg/m2)d
< 18.5	75 (32.1)	445 (31.1)	< 0.01
18.5–24.99	114 (48.7)	817 (57.0)
≥ 25.0	45 (19.2)	171 (11.9)
Mean ± SD	21.2 ± 4.2	20.7 ± 3.6	0.08
Smoking statusd
Never	179 (75.5)	959 (65.1)	< 0.01
Past	15 (6.3)	115 (7.8)
Current	43 (18.1)	399 (27.1)
Systolic blood pressure (mmHg)d
< 140	174 (75.0)	1,173 (81.5)	0.02
≥ 140	58 (25.0)	266 (18.5)
Mean ± SD	126.4 ± 22.9	122.8 ± 22.1	0.02
Diastolic blood pressure (mmHg)d
< 90	169 (72.8)	1,162 (80.8)	< 0.01
≥ 90	63 (27.2)	277 (19.2)
Mean ± SD	81.7 ± 14.3	79.1 ± 13.1	< 0.01
History of diabetesd
Yes	11 (4.6)	59 (4.0)	0.65
No	226 (95.4)	1,415 (96.0)
Well-water arsenic (µg/L)d
0.1–9	57 (24.1)	371 (25.3)	0.27
9.5–57	63 (26.6)	369 (25.2)
58–144	49 (20.7)	274 (25.5)
145–790	68 (28.7)	353 (24.1)
Mean ± SD	106.0 ± 125.3	92.8 ± 105.2	0.08
Urinary arsenic (µg/g creatinine)d
7–101	51 (22.3)	359 (25.3)	0.65
102–187	56 (24.5)	354 (25.0)
188–327	58 (25.3)	355 (25.1)
328–4306	64 (27.9)	349 (24.6)
Mean ± SD	280.8 ± 253.8	263.8 ± 273.3	0.38
Changes in urinary arsenic (µg/g creatinine)e	
Between baseline and the first follow-up	–64.7 (231.7)	–70.6 (237.0)	0.76
Between the first and second follow-up	–2.05 (201.0)	–0.04 (188.1)	0.89
ECG parameters (mean ± SD)
Heart rate	88.0 ± 16.9	75.3 ± 13.7	< 0.01
QTc	484.7 ± 45.0	420.4 ± 26.8	< 0.01
PR interval	136.9 ± 32.5	154.2 ± 25.6	< 0.01
QRS interval	97.4 ± 22.2	88.6 ± 25.1	< 0.01
aData were missing on QTc for 4 subjects; on BMI for 45 subjects; on education for 1 subject; on smoking status for 1 subject; and on systolic and diastolic blood pressure for 40 subjects. Data were also missing on well-water arsenic for 7 subjects and on baseline urinary arsenic for 64 subjects. bA QTc interval of ≥ 450 msec in men and ≥ 460 msec in women, respectively, was considered QTc prolongation. cp-Values were computed with the chi-square test or t-test. dCharacteristics assessed at baseline recruitment. eVisit-to-visit changes in urinary arsenic were estimated for original cohort only using visit-specific urinary creatinine–adjusted arsenic. For instance, changes in urinary arsenic between baseline and the first follow-up are the difference in urinary arsenic between the first follow-up and baseline (i.e., first follow-up urinary arsenic – baseline urinary arsenic).			

In the overall analysis, there was a positive association between continuous baseline well-water arsenic and QTc prolongation [model 3 OR = 1.17 (95% CI: 1.01, 1.35) for a 1-SD (108.7 µg/L) increase, *p*_trend_ = 0.04] (Table 2), but the categorical exposure model did not indicate a monotonic increase of ORs according to quartiles of well-water arsenic. The association with baseline urinary arsenic was similar [model 3 OR = 1.18 (95% CI: 0.97, 1.43) for a 1-SD (270.7 µg/g creatinine) increase], and the ORs increased monotonically with increasing quartiles of exposure; however, the trend *p*-value was not significant (*p*_trend_ = 0.10). In stratified analyses by sex, the prevalence of QTc prolongation was positively associated with baseline arsenic exposure in women, but not in men. The OR for QTc prolongation in association with the highest versus lowest quartile of baseline well-water arsenic (> 145 µg/L, mean 254.5 µg/L; and < 9 µg/L, mean 2.8 µg/L, respectively) was 1.61 (95% CI: 1.00, 2.58, model 3) in women and 0.76 (95% CI: 0.34, 1.69) in men. The adjusted ORs for a 1-SD increase in baseline well-water arsenic were 1.24 [(95% CI: 1.05, 1.47) in women; *p*_trend_ = 0.01], and 0.99 [(95% CI: 0.73, 1.33) in men; *p*_trend_ = 0.94]. The ORs for QTc prolongation comparing the highest and lowest quartiles of baseline urinary arsenic, and for continuous baseline urinary arsenic, were also positive in women (*p*_trend_ = 0.04), but not in men (*p*_trend_ = 0.60).

**Table 2:** Associations [OR (95% CI)] between baseline arsenic exposure variables and QTc prolongation measured during follow-up.

Variable	Arsenic exposure in quartilesa				Continuous arsenicb	pTrend
	Q1	Q2	Q3	Q4		
All						
Well-water arsenic (µg/L)						
n (cases/noncases)	57/371	63/369	49/374	68/353
Model 1c	1.00	1.11 (0.75, 1.64)	0.86 (0.57, 1.30)	1.24 (0.84, 1.82)	1.12 (0.98, 1.27)	0.10
Model 2d	1.00	1.08 (0.73, 1.59)	0.85 (0.56, 1.28)	1.24 (0.84, 1.83)	1.13 (0.99, 1.29)	0.08
Model 3e	1.00	1.10 (0.74, 1.63)	0.87 (0.57, 1.31)	1.31 (0.87, 1.96)	1.17 (1.01, 1.35)	0.04
Urinary arsenic (µg/g creatinine)						
n (cases/noncases)	51/359	56/354	58/355	64/349
Model 1c	1.00	1.09 (0.73, 1.65)	1.13 (0.75, 1.70)	1.22 (0.82, 1.81)	1.04 (0.91, 1.19)	0.53
Model 2d	1.00	1.13 (0.75, 1.70)	1.19 (0.79, 1.79)	1.30 (0.86, 1.97)	1.07 (0.93, 1.22)	0.35
Model 3e	1.00	1.14 (0.75, 1.72)	1.22 (0.81, 1.85)	1.42 (0.91, 2.21)	1.18 (0.97, 1.43)	0.10
Men
Well-water arsenic (µg/L)						
n (cases/noncases)	17/146	14/147	14/149	13/136
Model 1c	1.00	0.83 (0.39, 1.74)	0.83 (0.39, 1.74)	0.83 (0.39, 1.77)	1.01 (0.77, 1.33)	0.96
Model 2d	1.00	0.81 (0.38, 1.71)	0.86 (0.40, 1.82)	0.81 (0.38, 1.76)	1.02 (0.77, 1.36)	0.87
Model 3e	1.00	0.82 (0.39, 1.75)	0.85 (0.40, 1.82)	0.76 (0.34, 1.69)	0.99 (0.73, 1.33)	0.94
Urinary arsenic (µg/g creatinine)						
n (cases/noncases)	18/153	13/143	14/148	13/114
Model 1c	1.00	0.77 (0.36, 1.62)	0.80 (0.39, 1.67)	1.01 (0.47, 2.14)	1.00 (0.76, 1.32)	0.99
Model 2d	1.00	0.74 (0.35, 1.58)	0.82 (0.39, 1.73)	1.08 (0.49, 2.39)	1.05 (0.80, 1.38)	0.72
Model 3e	1.00	0.76 (0.36, 1.63)	0.83 (0.39, 1.76)	1.01 (0.44, 2.36)	0.86 (0.49, 1.51)	0.60
Women
Well-water arsenic (µg/L)						
n (cases/noncases)	40/225	49/222	35/225	55/217
Model 1c	1.00	1.24 (0.79, 1.96)	0.88 (0.54, 1.44)	1.43 (0.91, 2.23)	1.15 (0.99, 1.34)	0.06
Model 2d	1.00	1.19 (0.75, 1.88)	0.87 (0.53, 1.43)	1.46 (0.93, 2.30)	1.17 (1.00, 1.37)	0.05
Model 3e	1.00	1.22 (0.77, 1.93)	0.89 (0.54, 1.46)	1.61 (1.00, 2.58)	1.24 (1.05, 1.47)	0.01
Urinary arsenic (µg/g creatinine)						
n (cases/noncases)	33/206	43/211	44/207	51/235
Model 1c	1.00	1.28 (0.78, 2.10)	1.32 (0.81, 2.16)	1.35 (0.84, 2.17)	1.06 (0.91, 1.25)	0.46
Model 2d	1.00	1.32 (0.80, 2.17)	1.39 (0.85, 2.30)	1.48 (0.90, 2.41)	1.09 (0.93, 1.28)	0.30
Model 3e	1.00	1.31 (0.80, 2.16)	1.43 (0.87, 2.36)	1.69 (1.00, 2.86)	1.24 (1.01, 1.53)	0.04
aMean (range) of quartiles were Q1, 2.8 (0.1–9); Q2, 30.0 (9.5–57); Q3, 95.1 (58–144); and Q4, 254.5 (145–790) for well-water arsenic and Q1, 66.1 (7–101); Q2, 140.8 (102–187); Q3, 249.7 (188–327); and Q4, 606.3 (328–4306) for urinary arsenic. bORs for a 1-SD increase in well-water arsenic (108.7 µg/L) and urinary arsenic (270.7 µg/g creatinine). cAdjusted for sex and age (years); or for age in the subgroups of men and women. dAdjusted for model 1 variables plus BMI, smoking status (never, past, and current), and educational attainment (years). eAdjusted for model 2 variables plus changes in urinary arsenic (µg/g creatinine) between visits.						

Results from linear regression of QTc as a continuous dependent variable were consistent with the findings based on dichotomized QTc [see Supplemental Material, Table S2 (http://dx.doi.org/10.1289/ehp.1205197)]. For example, a 1-SD increase in well-water arsenic was associated with a 2.2-msec (95% CI: 0.3, 4.0) increase in mean QTc in the total population, and with 3.0-msec (95% CI: 0.3, 5.7) and 0.7-msec (95% CI: –1.5, 2.9) increases in mean QTc in women and men, respectively.

Adjusted estimates based on SG-adjusted urinary arsenic were consistent with those for creatinine-adjusted urinary arsenic. For example, the OR for QTc prolongation in relation to a 1-SD increase in baseline urinary arsenic was 0.94 (95% CI: 0.61, 1.44) in men (*p*_trend_ = 0.76) and 1.21 (95% CI: 1.01, 1.45) in women (*p*_trend_ = 0.04), respectively [see Supplemental Material, Table S3 (http://dx.doi.org/10.1289/ehp.1205197)]. Exclusion of participants with QRS ≥ 120 msec did not materially change the results (see Supplemental Material, Table S4). The positive associations remained similar in the subpopulation with ≥ 5 years of arsenic exposure (see Supplemental Material, Table S5). In the analyses of associations with urinary arsenic concentrations in the samples collected closest to the ECG measurement, effect estimates were consistent with, but weaker than, estimates from the main analysis (see Supplemental Material, Table S6). For instance, the adjusted OR for QTc prolongation in relation to a 1-SD increase in urinary arsenic concentration (216.8 µg/g creatinine) was 0.90 (95% CI: 0.60, 1.36) in men and 1.10 (95% CI: 0.97, 1.26) in women, respectively.

Prolongation of PR and QRS intervals was observed in 2.6% and 4.9% of the study population, respectively. There was no apparent association of either baseline well-water or baseline urinary arsenic with PR or QRS prolongation in the population as a whole (Table 3). Associations of baseline well-water and urinary arsenic with PR prolongation remained similar after excluding participants with QRS ≥ 120 msec (data not shown). Lastly, analyses of associations with prolonged heart rate–adjusted PR intervals generated very similar results (data not shown).

**Table 3:** Associations [OR (95%CI)] of 1-SD increases in baseline arsenic exposure variables with PR and QRS prolongation measured during follow-up.^a^

Variable	PR prolongation		QRS prolongation	
	OR (95% CI)	pTrend	OR (95% CI)	pTrend
Well-water arsenic (µg/L)b				
Model 1c	0.94 (0.68, 1.28)	0.67	1.15 (0.94, 1.41)	0.17
Model 2d	0.88 (0.63, 1.22)	0.44	1.12 (0.90, 1.39)	0.31
Model 3e	0.96 (0.68, 1.36)	0.83	1.09 (0.86, 1.37)	0.48
Urinary arsenic (µg/g creatinine)b				
Model 1c	0.72 (0.45, 1.14)	0.16	1.06 (0.87, 1.29)	0.56
Model 2d	0.69 (0.42, 1.11)	0.12	1.11 (0.91, 1.35)	0.29
Model 3e	0.80 (0.47, 1.37)	0.41	1.09 (0.76, 1.57)	0.63
aA PR interval of > 200 msec was considered PR prolongation; a QRS interval of ≥ 120 msec was considered QRS prolongation. bORs for a 1-SD increase in well-water arsenic (108.7 µg/L) and urinary arsenic (270.7 µg/g creatinine). n (cases/noncases) for PR prolongation analyses was 45/1,660 and 43/1,604 for well-water and urinary arsenic, respectively; n (cases/noncases) for QRS prolongation analyses was 84/1,622 and 79/1,569 for well-water and urinary arsenic, respectively. cAdjusted for sex and age (years). dAdjusted for model 1 variables plus BMI, smoking status (never, past, and current), and educational attainment (years). eAdjusted for model 2 variables plus changes in urinary arsenic (µg/g creatinine) between visits.				

## Discussion

In this study of arsenic exposure from drinking water and ECG abnormalities, we found a positive relationship between past arsenic exposure assessed at baseline and the QTc prolongation detected during follow-up. This association differed by sex and appeared to be present in women but not in men.

Several cross-sectional studies have assessed the association between arsenic exposure and QT interval. For instance, studies conducted in Bangladesh showed that the prevalence of prolonged QTc was significantly higher in arsenic-exposed individuals with skin lesions than in those without skin lesions or not exposed to arsenic (Ahmad et al. 2006). A study in Turkey found QTc intervals were longer in men (*n* = 40) chronically exposed to high levels of arsenic in drinking water (659 µg/L, range 422–1,066 µg/L) compared with men (*n* = 40) not exposed to arsenic (Yildiz et al. 2008). However, the sample size was small. A cross-sectional study of 300 adults (168 men and 145 women) in Inner Mongolia with well-water arsenic measured at the household level reported a monotonic relationship between well-water arsenic and QTc prolongation. However, the exposure categories were wide (≤ 21, 100–300, 430–690 μg/L) (Mumford et al. 2007). A recent study conducted in elderly men from the Normative Aging Study reported positive associations between toenail arsenic and QT duration (Mordukhovich et al. 2009). However, analyses were not conducted to assess effects of arsenic exposure on clinically significant levels of prolongation. More recently, a cross-sectional study of well-water arsenic measured at the village level in southwestern Taiwan reported a positive relationship and increased prevalence of QTc prolongation associated with arsenic concentrations > 700 µg/L in drinking water (Wang et al. 2009). Our prospective study, with the strengths of a large sample size (1,715 adults), a wide range of exposures (0.1–790 µg/L in drinking water, mean 95 µg/L), and exposure data at the individual level based on well-water arsenic and urinary arsenic concentrations at baseline and follow-up, adds to the weight of the evidence suggesting adverse effects of chronic arsenic exposure at moderate levels on QTc prolongation and that the effects were mostly limited to women. Total urinary arsenic has previously been shown to correlate well with well-water arsenic in our study population (with a correlation coefficient of > 0.70) (Ahsan et al. 2007). Associations with urinary arsenic in samples collected closest to the ECG measurement were weaker than associations with urinary arsenic concentrations at baseline that were adjusted for changes in concentrations during follow-up, whereas the analyses restricted to those with a more complete exposure history prior to baseline were similar to associations in the study population as a whole. Together, these data suggest that the effect on QTc prolongation was related to past and persistent arsenic exposure from drinking water measured at baseline. Future longitudinal studies with repeated ECG measurements would be valuable in assessing the influence of longitudinal changes in exposure on changes in QTc.

In our previous cohort analyses of CVD mortality, we observed a positive relationship between well-water arsenic and overall CVD mortality, with hazard ratios ranging from 1.22 to 1.92 for the three highest quartiles of well-water arsenic compared with the lowest quartile, and the association was similar in men and women (Chen Y et al. 2011b). However, in the present study, QTc prolongation was significantly associated with the highest quartile of exposure in women only [OR = 1.61 (95% CI: 1.00, 2.58)]. Given that CVD is a broad set of diseases and condition, QTc prolongation might not be a main driver of the overall CVD mortality. QTc prolongation may be a more critical intermediate endpoint for higher levels of exposure and for subtypes of CVD, such as coronary heart disease. The data on different end points support the idea that the cardiovascular effects of arsenic exposure may differ according to dose and subtypes of CVD, and may be increased in susceptible subsets of the population.

We found that the positive association between arsenic exposure and QTc prolongation was only present in women. Among men, the ORs were below the null for categorical exposures, but were imprecise and not significantly negative. Associations with arsenic as a continuous variable were consistent with the null, and overall, the findings for men did not support an effect of arsenic exposure on ECG abnormalities, in contrast with the findings for women. Data from the study in Inner Mongolia (Mumford et al. 2007) also indicated a potentially greater susceptibility in women. Several *in vitro* and *in vivo* studies have shown that female sex is a strong risk factor for drug-induced long QT interval and cardiac arrhythmias (Liu et al. 1998; Lu et al. 2001). It is well known that females have longer mean QT intervals than males (Schwartz 2000). Women are also more susceptible to develop torsades de pointes during administration of drugs that prolong QT (Lehmann et al. 1996; Makkar et al. 1993; Reinoehl et al. 1996; Zareba et al. 1995). Although the mechanisms for the differences in QT intervals in men and women are uncertain, previous studies suggest that sex hormones and differences in cardiac ionic channels or serum potassium levels may contribute to sex-specific differences in ventricular repolarization (Drici et al. 1996; Fukui et al. 2003; Liu et al. 1998). Future studies are needed to investigate the underlying mechanisms of the sex differences in arsenic-induced ECG abnormalities.

Potential mechanisms by which arsenic might induce QT-interval prolongation are also unclear. Arsenic may induce abnormalities of cardiac repolarization by increasing cardiac calcium currents and reducing surface expression of cardiac potassium channel genes such as *hERG* (human ether-a-go-go–related gene) and *KCNQ1* (potassium voltage-gated channel, KQT-like subfamily, member 1) (Curran et al. 1995; Wang et al. 1996). Arsenic interferes with hERG trafficking to the cell surface by inhibiting hERG-chaperone complexes and increasing calcium currents as shown in a series of biochemical and electrophysiological experiments *in vitro* (Ficker et al. 2004). Other possible mechanisms of arsenic-induced QT prolongation include alterations in DNA repair and methylation, generation of reactive oxygen species, and induction of cardiomyocyte apoptosis (Flora et al. 2007; Zhao et al. 2008).

The study has several limitations. First, our study population consisted of relatively few overweight individuals, possessing a mean BMI of 20.8. Thus, the findings may not be generalizable to other populations with a different nutritional profile. However, our data were consistent with those of Mumford et al. (2007) from a population in Inner Mongolia with a completely different ethnic and cultural background and a better nutritional status (average BMI > 22.5), suggesting that the findings may be generalizable to other populations. Second, we did not evaluate potential effect modification by specific nutritional factors that may play a role in prevention of CVD, such as antioxidants (Palace et al. 1999; Willcox et al. 2008). However, no evidence of interaction between arsenic and antioxidant intake was found for QTc interval in the study of a U.S. general population (Mordukhovich et al. 2009). Third, the study was conducted in participants who were referred for ECG examinations because of potential health problems. However, because of our comprehensive follow-up mechanism, ECG was performed for individuals with and without risk of heart conditions. The distributions of lifestyle, demographic, and arsenic exposure variables suggest that the study population was similar to the overall cohort.

## Conclusions

We found a positive association between past arsenic exposure in a population with long-term exposures at moderate levels and QT-interval prolongation measured on average 5.9 years after baseline and the association was present in women but not in men. Our finding may help explain the increased mortality from CVD in humans exposed to arsenic from drinking water. ECG analysis of the QT interval may be useful for early detection of cardiac toxicity induced by arsenic exposure and for evaluation of populations at high risk for cardiovascular events.

## Supplemental Material

(389 KB) PDF
