# Supplementary material for: Arsenic Exposure from Drinking Water and QT-Interval Prolongation: Results from the Health Effects of Arsenic Longitudinal Study
Source: Environ Health Perspect. 2013 Feb 5;121(4):427–32. doi: 10.1289/ehp.1205197 (PMC3620737; doi:10.1289/ehp.1205197)
Supplement: (389 KB) PDF [file ehp.1205197.s001.pdf]

Supplemental Material

Arsenic Exposure from Drinking Water and QT-Interval Prolongation: Results from the Health Effects of Arsenic Longitudinal Study

Yu Chen, Fen Wu, Faruque Parvez, Alauddin Ahmed, Mahbub Eunus, Tyler R. McClintock, Tazul Islam Patwary, Tariqul Islam, Anajan Kumar Ghosal, Shahidul Islam, Rabiul Hasan, Diane Levy, Golam Sarwar, Vesna Slavkovich, Alexander van Geen, Joseph H. Graziano, and Habibul Ahsan

Table of Contents:

Table S1. Characteristics of participants in the present study and participants in the overall cohort study [n (%) or mean ± SD]........ 2

Table S2. Associations [coefficient (95% CI)] between baseline arsenic exposure variables and QTc interval (ms) measured during follow-up 3

Table S3. Associations [OR (95% CI)] between baseline urinary specific gravity adjusted arsenic and QTc prolongation measured during follow-up........ 4

Table S4. Associations [OR (95% CI)] between baseline arsenic exposure variables and QTc prolongation measured during follow-up........ 5

Table S5. Associations [OR (95% CI)] between baseline arsenic exposure variables and QTc prolongation, in subpopulation with longer-term arsenic exposure (≥ 5 years) 6

Table S6. Associations [OR (95% CI)] between urinary arsenic closest to the ECG measurement and QTc prolongation measured during follow-up........ 7

Table S1. Characteristics of participants in the present study and participants in the overall cohort study [n (%) or mean ± SD]

	Present study participants (*n* = 1,715)	Overall cohort participants (*n* = 20,033)
Sex		
Women	1074 (62.6)	11870 (59.3)
Men	641 (37.4)	8163 (40.7)
Age (years) at baseline	40.4 ± 9.8	36.9 ± 10.4
BMI (kg/m^2)	20.8 ± 3.7	19.8 ± 3.2
Education (years)	3.6 ± 3.9	3.5 ± 3.8
Smoking status		
Never	1139 (66.4)	13510 (67.4)
Past	130 (7.6)	1255 (6.3)
Current	445 (26.0)	5260 (26.3)
Systolic blood pressure (mm Hg)	123.3 ± 22.3	116.6 ± 17.2
Diastolic blood pressure (mm Hg)	79.4 ± 13.3	74.9 ± 11.2
Well water arsenic (µg/L)	95.1 ± 108.7	81.7 ± 105.9
Well use duration (years)	8.0 ± 5.6	7.6 ± 5.1
Urinary arsenic (µg/L)	124.1 ± 136.6	118.7 ± 144.5
Urinary creatinine (mg/dL)	56.0 ± 44.5	53.8 ± 42.6
Urinary arsenic (µg/g creatinine)	266.6 ± 270.7	259.2 ± 285.3

Table S2. Associations [coefficient (95% CI)] between baseline arsenic exposure variables and QTc interval (ms) measured during follow-up

Arsenic exposure variable	Arsenic exposure in quartiles[a] 1	2	3	4	Continuous arsenic[b]	*p*-trend
All						
Well water arsenic (µg/L)						
n	428	432	424	424		
Model 3[c]	Ref	-0.45 (-5.26-4.36)	2.01 (-2.86-6.88)	4.66 (-0.44-9.76)	2.16 (0.28-4.04)	0.02
Urinary arsenic (µg/g creatinine)						
n	411	410	414	415		
Model 3[c]	Ref	-2.19 (-6.99-2.61)	2.80 (-2.04-7.63)	2.32 (-3.01-7.64)	2.02 (-0.50-4.54)	0.12
Men						
Well water arsenic (µg/L)						
n	163	161	163	152		
Model 3[c]	Ref	-1.69 (-7.30-3.93)	-0.96 (-6.60-4.68)	0.58 (-5.34-6.51)	0.71 (-1.52-2.93)	0.53
Urinary arsenic (µg/g creatinine)						
n	172	156	162	129		
Model 3[c]	Ref	-3.73 (-9.30-1.84)	1.25 (-4.30-6.81)	-1.32 (-7.80-5.16)	0.12 (-3.59-3.82)	0.95
Women						
Well water arsenic (µg/L)						
n	265	271	261	272		
Model 3[c]	Ref	0.36 (-6.57-7.28)	4.15 (-2.90-11.2)	7.32 (-0.04-14.7)	3.01 (0.32-5.70)	0.03
Urinary arsenic (µg/g creatinine)						
n	239	254	252	286		
Model 3[c]	Ref	-1.35 (-8.36-5.66)	3.97 (-3.12-11.1)	4.93 (-2.68-12.5)	2.94 (-0.39-6.27)	0.08

[a]Mean (range) of quartiles were 2.8 (0.1–9), 30.0 (9.5–57), 95.1 (58–144), and 254.5 (145–790) for well water arsenic and 66.1 (7–101), 140.8 (102–187), 249.7 (188–327), and 606.3 (328–4306) for urinary arsenic.
[b]ORs for a 1-SD increase in well water arsenic (108.7 µg/L) and urinary arsenic (270.7 µg/g creatinine).
[c]Adjusted for sex, age, body mass index, smoking status, educational attainment, and changes in urinary arsenic (µg/g creatinine) between visits; or for age, body mass index, smoking status, educational attainment, and changes in urinary arsenic (µg/g creatinine) between visits in the subgroups of men and women.

Table S3. Associations [OR (95% CI)] between baseline urinary specific gravity adjusted arsenic and QTc prolongation measured during follow-up

	Arsenic exposure in quartiles[a]					
	1	2	3	4	Continuous arsenic[b]	*p*-trend
All						
n (cases/noncases)	56/352	51/353	55/354	66/339		
Model 1[c]	1.00	0.93 (0.62, 1.40)	1.00 (0.67, 1.50)	1.23 (0.84, 1.82)	1.12 (0.98, 1.27)	0.10
Model 2[d]	1.00	0.95 (0.63, 1.43)	1.03 (0.68, 1.54)	1.30 (0.88, 1.92)	1.13 (1.00, 1.32)	0.06
Model 3[e]	1.00	0.96 (0.63, 1.44)	1.03 (0.68, 1.54)	1.30 (0.85, 1.98)	1.16 (0.99, 1.37)	0.07
Men						
n (cases/noncases)	19/127	6/151	14/148	19/125		
Model 1[c]	1.00	0.27 (0.11, 0.70)	0.64 (0.31, 1.32)	1.04 (0.52, 2.06)	1.10 (0.83, 1.46)	0.52
Model 2[d]	1.00	0.29 (0.11, 0.74)	0.68 (0.32, 1.42)	1.28 (0.62, 2.61)	1.18 (0.89, 1.57)	0.26
Model 3[e]	1.00	0.28 (0.11, 0.74)	0.62 (0.29, 1.32)	1.06 (0.47, 2.38)	0.94 (0.61, 1.44)	0.76
Women						
n (cases/noncases)	37/225	45/202	41/206	47/214		
Model 1[c]	1.00	1.35 (0.84, 2.18)	1.21 (0.75, 1.96)	1.34 (0.83, 2.14)	1.12 (0.97, 1.30)	0.13
Model 2[d]	1.00	1.36 (0.85, 2.19)	1.24 (0.76, 2.02)	1.38 (0.86, 2.22)	1.13 (0.98, 1.31)	0.09
Model 3[e]	1.00	1.38 (0.85, 2.22)	1.26 (0.77, 2.05)	1.41 (0.85, 2.34)	1.21 (1.01, 1.45)	0.04

[a]Mean (range) of quartiles for urinary arsenic (µg/L) were 27.8 (5.2–42.9), 64.2 (43.3–85.3), 117.6 (85.8–156.9), and 294.1 (157.3–1150.5).
[b]ORs for a 1-SD increase in urinary arsenic (128.1 µg/L).
[c]Adjusted for sex and age; or for age in the subgroups of men and women.
[d]Adjusted for Model 1 variables plus body mass index, smoking status (never, past, and current), and educational attainment.
[e]Adjusted for Model 2 variables plus changes in urinary specific gravity adjusted arsenic (µg/L) between visits.

Table S4. Associations [OR (95% CI)] between baseline arsenic exposure variables and QTc prolongation measured during follow-up[a]

Arsenic exposure variable	Arsenic exposure in quartiles[b] 1	2	3	4	Continuous arsenic[c]	*p*-trend
**All**						
Well water arsenic (µg/L)						
n (cases/noncases)	46/362	57/356	42/359	57/341		
Model 3[d]	1.00	1.24 (0.81, 1.89)	0.93 (0.59, 1.47)	1.44 (0.93, 2.24)	1.15 (0.98, 1.35)	0.09
Urinary arsenic (µg/g creatinine)						
n (cases/noncases)	51/359	56/354	58/355	64/349		
Model 3[d]	1.00	1.04 (0.66, 1.62)	1.29 (0.83, 2.00)	1.42 (0.88, 2.28)	1.20 (0.99, 1.47)	0.07
**Men**						
Well water arsenic (µg/L)						
n (cases/noncases)	12/138	11/137	10/137	10/127		
Model 3[d]	1.00	0.92 (0.39, 2.18)	0.91 (0.37, 2.19)	0.83 (0.33, 2.07)	0.82 (0.55, 1.21)	0.82
Urinary arsenic (µg/g creatinine)						
n (cases/noncases)	15/146	7/134	13/138	8/102		
Model 3[d]	1.00	0.51 (0.20, 1.29)	0.94 (0.42, 2.10)	0.75 (0.27, 2.06)	0.67 (0.32, 1.40)	0.60
**Women**						
Well water arsenic (µg/L)						
n (cases/noncases)	34/224	46/219	32/222	47/214		
Model 3[d]	1.00	1.36 (0.84, 2.21)	0.97 (0.57, 1.64)	1.71 (1.04, 2.84)	1.25 (1.05, 1.50)	0.01
Urinary arsenic (µg/g creatinine)						
n (cases/noncases)	29/203	38/211	40/204	46/233		
Model 3[d]	1.00	1.30 (0.77, 2.20)	1.48 (0.87, 2.51)	1.80 (1.04, 3.11)	1.28 (1.04, 1.59)	0.02

[a]Participants with a QRS interval ≥ 120 ms were excluded from the analyses.
[b]Mean (range) of quartiles were 2.8 (0.1–9), 30.0 (9.5–57), 95.1 (58–144), and 254.5 (145–790) for well water arsenic and 66.1 (7–101), 140.8 (102–187), 249.7 (188–327), and 606.3 (328–4306) for urinary arsenic.
[c]ORs for a 1-SD increase in well water arsenic (108.7 µg/L) and urinary arsenic (270.7 µg/g creatinine).
[d]Adjusted for sex, age, body mass index, smoking status, educational attainment, and changes in urinary arsenic (µg/g creatinine) between visits; or for age, body mass index, smoking status, educational attainment, and changes in urinary arsenic (µg/g creatinine) between visits in the subgroups of men and women.

Table S5. Associations [OR (95% CI)] between baseline arsenic exposure variables and QTc prolongation, in subpopulation with longer-term arsenic exposure (≥ 5 years)

	Well water arsenic (µg/L)[a]		Urinary arsenic (µg/g creatinine)[a]	
	Continuous arsenic[b]	*p*-trend	Continuous arsenic[b]	*p*-trend
All[c]				
Model 1[d]	1.12 (0.95, 1.31)	0.18	1.04 (0.89, 1.21)	0.66
Model 2[e]	1.12 (0.95, 1.32)	0.17	1.05 (0.90, 1.23)	0.52
Model 3[f]	1.17 (0.97, 1.40)	0.09	1.17 (0.93, 1.48)	0.18
Men[c]				
Model 1[d]	0.93 (0.63, 1.35)	0.69	0.95 (0.66, 1.37)	0.79
Model 2[e]	0.93 (0.62, 1.38)	0.70	0.99 (0.69, 1.43)	0.95
Model 3[f]	0.89 (0.58, 1.35)	0.58	0.85 (0.42, 1.72)	0.65
Women[c]				
Model 1[d]	1.17 (0.98, 1.40)	0.09	1.06 (0.89, 1.27)	0.50
Model 2[e]	1.19 (0.99, 1.43)	0.07	1.09 (0.91, 1.31)	0.36
Model 3[f]	1.25 (1.02, 1.52)	0.03	1.24 (0.97, 1.59)	0.09

[a]Mean values of well water arsenic and urinary arsenic were 93.0 µg/L and 270.7 µg/g creatinine in all; 88.8 and 237.0 in men; 95.5 and 280.9 in women, respectively. Mean duration of exposure with known arsenic concentrations was 10.1 years in all, 10.7 years in men, and 9.7 years in women.
[b]ORs for a 1-SD increase in well water arsenic (108.7 µg/L) and urinary arsenic (270.7 µg/g creatinine).
[c]*n* (cases/non-cases) was 164/969 and 159/938 for well water arsenic and urinary arsenic, respectively, in all; 38/376 and 38/365, respectively, in men; 126/593 and 121/573, respectively, in women.
[d]Adjusted for sex and age; or for age in the subgroups of men and women.
[e]Adjusted for Model 1 variables plus body mass index, smoking status (never, past, and current), and educational attainment.
[f]Adjusted for Model 2 variables plus changes in urinary arsenic (µg/g creatinine) between visits.

Table S6. Associations [OR (95% CI)] between urinary arsenic closest to the ECG measurement and QTc prolongation measured during follow-up[a]

	Urinary arsenic in quartiles[b]					
	1	2	3	4	Continuous arsenic[c]	*p*-trend
**All**						
n (cases/non-cases)	56/365	53/368	53/368	72/346		
Model 1[d]	1.00	0.90 (0.60, 1.35)	0.90 (0.60, 1.35)	1.27 (0.86, 1.86)	1.07 (0.95, 1.21)	0.26
Model 2[e]	1.00	0.94 (0.62, 1.42)	0.94 (0.63, 1.43)	1.30 (0.88, 1.92)	1.08 (0.95, 1.22)	0.23
**Men**						
n (cases/non-cases)	22/162	10/143	12/143	13/121		
Model 1[d]	1.00	0.52 (0.24, 1.14)	0.63 (0.30, 1.32)	0.82 (0.40, 1.70)	0.89 (0.59, 1.33)	0.56
Model 2[e]	1.00	0.53 (0.24, 1.17)	0.65 (0.30, 1.39)	0.84 (0.40, 1.79)	0.90 (0.60, 1.36)	0.61
**Women**						
n (cases/non-cases)	34/203	43/225	41/225	59/225		
Model 1[d]	1.00	1.14 (0.70, 1.86)	1.08 (0.66, 1.77)	1.56 (0.98, 2.47)	1.10 (0.97, 1.25)	0.16
Model 2[e]	1.00	1.21 (0.74, 1.98)	1.13 (0.69, 1.87)	1.62 (1.00, 2.60)	1.10 (0.97, 1.26)	0.14

[a]Urinary arsenic closest to ECG measurement was baseline urinary arsenic for expansion cohort participants (*n* = 273), urinary arsenic at the second follow-up for original cohort participants whose ECGs were performed after the second follow-up, or within the 6 months before the second follow-up (*n* = 1,402), and urinary arsenic at the first follow-up for participants (*n* = 38) whose ECG was performed > 6 months before the second follow-up.
[b]Mean (range) of quartiles for urinary arsenic were 57.5 (0–83), 112.2 (84–145), 196.1 (146–260), and 476 (261–3419).
[c]ORs for a 1-SD increase in urinary arsenic (216.8 µg/g creatinine).
[d]Adjusted for sex and age; or for age in the subgroups of men and women.
[e]Adjusted for Model 1 variables plus body mass index, smoking status (never, past, and current), and educational attainment.
